# Revisiting tissue vs. intravascular congestion: a framework for targeted decongestion in patients with acute decompensated heart failure

**DOI:** 10.1093/ehjcr/ytaf346

**Published:** 2025-07-18

**Authors:** Takeshi Kitai, Kitae Kim, Piotr Nikodem Rudziński

**Affiliations:** Department of Heart Failure and Transplantation, National Cerebral and Cardiovascular Centre, 6-1 Kishibe Shinmachi, Osaka 564-8565, Japan; Department of Cardiovascular Medicine, Kobe City Medical Center General Hospital, 2-1-1 Minatojima-minamimachi, Chuo-ku, Kobe 650-0047, Japan; Department of Coronary and Structural Heart Diseases, the Cardinal Stefan Wyszyński, National Institute of Cardiology, Alpejska 42, Warsaw 04-628, Poland

**This editorial refers to ‘Therapeutic role of venous leg compression in worsening heart failure with predominant extravascular congestion: a case report’, by S. Mínguez *et al*., doi:https://doi.org/10.1093/ehjcr/ytaf343.**

Congestion is a hallmark feature and a major driver of hospitalization in patients with acute decompensated heart failure (ADHF). While most patients exhibit both intravascular and tissue congestion, the relative predominance of each varies and significantly influences therapeutic response. Currently, the mainstay of decongestion therapy in ADHF patients remains loop diuretics, which promote natriuresis and reduce circulatory blood volume, thereby alleviating intravascular congestion.^1–3^ However, by reducing intravascular stressed volume, this approach may trigger activation of the renin–angiotensin–aldosterone system and potentially lead to worsening renal function.^4^

During the course of decongestive therapy in patients with ADHF, two distinct scenarios may result in intravascular hypovolaemia. The first is a straightforward over-diuretic state leading to volume depletion. The second—and more challenging—scenario arises when intravascular volume is depleted despite persistent tissue congestion. This mismatch occurs when the rate of diuresis exceeds the capacity for interstitial-to-intravascular fluid translocation. In clinical practice, it is often this latter group of patients in whom decongestion becomes most difficult. In such cases, the balance between intravascular volume reduction and vascular refill becomes critical. When this balance is disrupted, additional diuretics may worsen hypotension and renal dysfunction without achieving adequate decongestion. Therapeutic strategies that promote fluid redistribution rather than simple volume removal may be more physiologically appropriate. Therefore, it is essential not only to distinguish between intravascular and tissue congestion separately at baseline but also to reassess them throughout the course of treatment.

A meta-analysis revealed that the co-administration of loop diuretics with albumin may enhance diuresis, particularly in patients with baseline albumin levels below 2.5 g/dL or those with impaired renal function, despite substantial heterogeneity in treatment response.^5^ Infusion of hypertonic saline can increase intravascular osmotic pressure, potentially promoting vascular refill from the interstitial and intracellular compartments and enhancing renal perfusion, thereby augmenting the diuretic response to loop diuretics. There are data showing efficacy and safety of hypertonic saline in addition to loop diuretic therapy compared with loop diuretics alone in patients with ADHF. However, there is a concern that sodium loading may exacerbate further congestion in ADHF patients, and evidence from randomized clinical trials is still lacking.^6^

Among pharmacologic options, vasopressin V2 receptor antagonists such as tolvaptan induce aquaresis, enhancing plasma osmolality and drawing fluid from the interstitial to the intravascular space. A randomized controlled study investigating the effects of tolvaptan in patients hospitalized with ADHF demonstrated that tolvaptan did not adversely affect blood pressure, heart rate, or renal function.^7^ These findings support the hypothesis that the increase in plasma osmolality induced by tolvaptan may facilitate the translocation of interstitial fluid into the intravascular space. Similarly, sodium-glucose transport protein 2 (SGLT2) inhibitors suppress glucose reabsorption, which is coupled with concurrent sodium transport in the proximal convoluted tubule, thereby promoting natriuresis. In addition, glucose that is not reabsorbed due to SGLT2 inhibition flows into the distal nephron. As the concentration of glucose in the tubular fluid increases, the osmotic gradient between the tubular fluid and the interstitium is reduced, thereby decreasing passive water reabsorption, primarily in the collecting duct, resulting in osmotic diuresis. A previous study employed a mathematical model to illustrate that free water clearance leads to a greater reduction in interstitial fluid volume compared with blood volume. In this model, the loss of free water increases plasma sodium concentration, promoting the movement of fluid from the interstitial space into the circulation. As sodium concentration in the interstitial space rises, sodium is redistributed towards peripheral compartments (‘sequestrated’), where it becomes osmotically inactive. Consequently, the interstitial fluid volume decreases while the sodium concentration remains relatively constant. Data on plasma and urinary sodium and water from healthy subjects administered an SGLT2 inhibitor were analysed using this model, demonstrating that SGLT2 inhibition resulted in a two-fold greater reduction in interstitial fluid volume compared with blood volume.^8^ Thus, aquaresis induced by tolvaptan or SGLT2 inhibitors may alleviate congestion by preferentially removing interstitial fluid while preserving blood pressure and organ perfusion.

In the current issue of *European Heart Journal Case Reports*, Mínguez *et al*. presented a compelling case of a 50-year-old woman with AL amyloidosis and ADHF, marked by severe peripheral oedema but no signs of intravascular congestion.^9^ Treatment combining subcutaneous loop diuretics with venous leg compression (VLC) resulted in effective tissue decongestion and enhanced intravascular refill. This case highlights two important considerations. First, increasing preload through interstitial-to-intravascular fluid shift may be beneficial in selected patients, challenging the conventional view that preload augmentation is generally considered contraindicated in this setting. Second, VLC may serve as a low-risk, non-pharmacological adjunct for managing tissue congestion, particularly when traditional diuretics are limited by the absence of intravascular volume overload.

Venous leg compression, including multilayered bandaging, manual lymphatic drainage, and compression stockings, has long been used in the management of interstitial fluid accumulation resulting from impaired lymphatic function and venous circulation, such as in peripheral lymphoedema and venous insufficiency. Venous leg compression can increase interstitial hydrostatic pressure, thereby promoting the movement of fluid from the interstitial space into the intravascular compartment. Although the splanchnic veins, which comprise the abdominal compartment of the venous circulation, have the greatest capacitance among all venous beds,^10^ the veins in the lower limbs may also contribute, to some extent, to venous reservoir function. Thus, the use of VLC in patients with ADHF remains controversial due to concerns that it may increase right-sided and subsequently left-sided preload, potentially worsening right and left ventricular functions. There are conflicting data regarding the haemodynamic effects of VLC in patients with heart failure. Some studies reported no significant impact on filling pressures as assessed by Doppler echocardiography or increases in right heart pressure or pulmonary artery wedge pressure without associated clinical consequences.^11,12^ In contrast, another study demonstrated significant increases in right atrial pressure, pulmonary artery wedge pressure, and systemic vascular resistance, without a corresponding increase in cardiac index, which may suggest potentially unfavourable haemodynamic effects.^13^ Therefore, VLC may be considered in the selected patients when right atrial pressure is not elevated and should be used with caution in patients with heart failure and severely impaired right or left ventricular function.

Effective management of ADHF requires more than just volume removal—it demands a nuanced understanding of how fluid is distributed across vascular and interstitial compartments. Differentiating between intravascular and tissue congestion by integrating physical findings, biomarkers, and echocardiographic parameters,^14,15^ and recognizing when these two forms are mismatched, is critical for guiding therapy (*Figure 1*). Further studies are needed to better understand the efficacy and safety of fluid redistribution therapies, including VLC, in this complex clinical setting. Reframing congestion as a problem of distribution rather than volume may ultimately reshape how we treat the most challenging patients.

**Figure 1:**
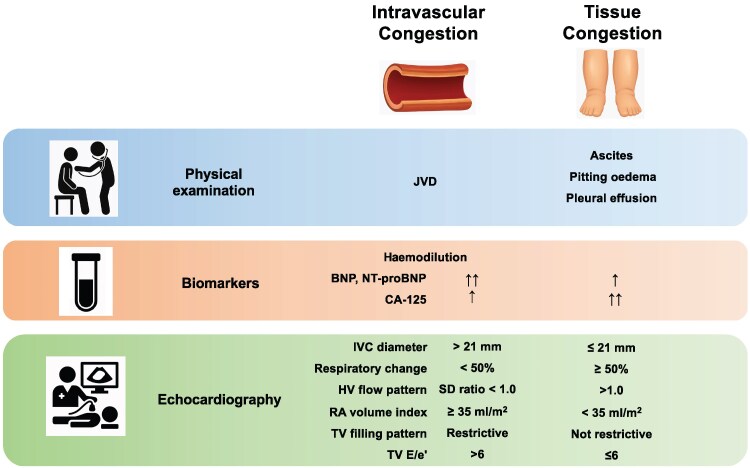
Clinical and diagnostic markers distinguishing intravascular and tissue congestion. This figure summarizes key physical findings, biomarkers, and echocardiographic parameters used to differentiate between intravascular and tissue congestion in patients with acute decompensated heart failure. Integrated assessment of these variables is essential for guiding targeted decongestion strategies. JVD, jugular vein distention; BNP, B-type natriuretic peptide; NT-proBNP, N-terminal pro-brain natriuretic peptide; CA 125, carbohydrate antigen 125; IVC, inferior vena cava; HV, hepatic vein; RA, right atrial; and TV, tricuspid valve.

## Lead author biography

Takeshi Kitai has served as a director in the section of Heart Failure and Structural Heart Disease, Department of Cardiovascular Medicine, National Cerebral and Cardiovascular Center in Japan, holding the tile of MD, PhD, FHSA, and FESC. He completed his clinical research training in heart failure at Cleveland Clinic in the US. Dr Kitai's specialty interests include cardiomyopathy, heart failure including heart transplantation and mechanical circulatory assist devices, valvular heart disease, and structural heart intervention.
